# Petrol Dermatitis

**Published:** 1918-09

**Authors:** 


**Petrol Dermatitis.** By Surgeon G.B. PAGE. M.B.R.N. *The Practitioner*, London, May 1918.

Page discusses the dermatitis among aviators due to the prolonged contact of petrol-soaked clothing with the skin. He states that it is a frequent condition, particularly after aeroplane crashes, when the aviator may be pinned by the wreckage beneath a bursted tank, and so dazed or injured that he does not remove his petrol-soaked clothing for some time after the accident.

This petrol dermatitis resembles a burn or scald of the first and second degree. There is erythema, sometimes vesication, with burning pain of varying degrees. The area involved is often large, e.g., both legs, thighs, and feet.

The treatment recommended is a lead lotion applied on lint or zinc-carbolic lotion (zinc oxid drs. 3, suspended in glycerin, oz. 1, in 1 o/o carbolic acid solution to oz. 8). This may be followed by a simple dressing powder after the symptoms subside, a process which fortunately is very rapid. The affected parts should be uncovered in weather, or else a cradle used to support the bed clothes.

In order to prevent petrol dermatitis after an accident, if the clothing is soaked with petrol, it should be removed as soon as circumstances will permit.

